# Identification, Cloning and Heterologous Expression of the Gene Cluster Directing RES-701-3, -4 Lasso Peptides Biosynthesis from a Marine *Streptomyces* Strain

**DOI:** 10.3390/md18050238

**Published:** 2020-05-01

**Authors:** Daniel Oves-Costales, Marina Sánchez-Hidalgo, Jesús Martín, Olga Genilloud

**Affiliations:** Fundación MEDINA, Centro de Excelencia en Investigación de Medicamentos Innovadores en Andalucía, Avda del Conocimiento 34, 18016 Armilla (Granada), Spain; marina.sanchez@medinaandalucia.es (M.S.-H.); jesus.martin@medinaandalucia.es (J.M.); olga.genilloud@medinaandalucia.es (O.G.)

**Keywords:** lasso peptide, RES-701-3, RES-701-4, genome mining, biosynthetic gene cluster, *Streptomyces caniferus*

## Abstract

RES-701-3 and RES-701-4 are two class II lasso peptides originally identified in the fermentation broth of *Streptomyces* sp. RE-896, which have been described as selective endothelin type B receptor antagonists. These two lasso peptides only differ in the identity of the C-terminal residue (tryptophan in RES-701-3, 7-hydroxy-tryptophan in RES-701-4), thus raising an intriguing question about the mechanism behind the modification of the tryptophan residue. In this study, we describe the identification of their biosynthetic gene cluster through the genome mining of the marine actinomycete *Streptomyces caniferus* CA-271066, its cloning and heterologous expression, and show that the seven open reading frames (ORFs) encoded within the gene cluster are sufficient for the biosynthesis of both lasso peptides. We propose that ResE, a protein lacking known putatively conserved domains, is likely to play a key role in the post-translational modification of the C-terminal tryptophan of RES-701-3 that affords RES-701-4. A BLASTP search with the ResE amino acid sequence shows the presence of homologues of this protein in the genomes of eight other *Streptomyces* strains, which also harbour the genes encoding the RES-701-3, -4 precursor peptide, split-B proteins and ATP-dependent lactam synthetase required for the biosynthesis of these compounds.

## 1. Introduction

Lasso peptides are a class of ribosomally synthesised and post-translationally modified peptides (RiPPs) natural products produced by the bacterial domain [1,2]. They have been shown to possess a wide range of biological properties, including antimicrobial activity against Gram-negative pathogens in the case of capistruin [3] and microcin J25 [4], antimicrobial activity against Gram-positive pathogens such as that exerted by siamycin-I [5] and the inhibition of HIV replication displayed in cell cultures by RP71955 [6]. A striking feature of lasso peptides, which sets them apart from other RiPPs, is their unusual topology. All of them display a macrolactam ring comprising seven to nine residues, formed between the N-terminal α-amino group and the β- or γ-carboxyl group of aspartic or glutamic acid, with the remaining C-terminal peptide tail threaded through the ring. Additionally, the topology of some lasso peptides can be further modified by the presence of one or two disulfide bridges (class III and I lasso peptides respectively), although most of the known lasso peptides contain none (class II lasso peptides) [2]. The vast majority of lasso peptides have been isolated from terrestrial microorganisms; however, in recent years, some have been obtained from marine bacteria, including sungsanpin and aborycin [7,8]. 

The advent of genomics in recent years has dramatically changed the landscape of lasso peptide discovery and research. Whereas, in the past, lasso peptides were serendipitously discovered employing untargeted bioactivity-guided approaches, now the field is flourishing with genome-mining based targeted strategies [9]. These strategies require the bioinformatic analysis of genomic information, and numerous platforms able to do so have been developed in recent years, such as antiSMASH (antibiotics and secondary metabolites analysis shell) [10], BAGEL (bacteriocin genome mining tool) [11], RiPPMiner (ribosomally synthesized post-translationally modified peptides miner) [12], PRISM (prediction informatics for secondary metabolomes) [13] and RODEO (rapid ORF description and evaluation online) [14]. The increasing number of lasso peptide biosynthetic gene clusters identified and characterised has also led to a better understanding of their biosynthesis. The minimum biosynthetic gene cluster comprises a gene encoding a precursor peptide (termed A), containing the leader and core peptides; a cysteine protease (termed B), which is involved in the maturation step and the assembly of the knot-like structure; and an ATP-dependent lactam synthetase (termed C), which is also required for the maturation step and the formation of the 7–9 residue macrolactam ring. Some lasso peptide gene clusters display a split B gene, with the B1 gene encoding for the N-terminal region and the B2 gene for the C-terminal part of a B protein encoded by an intact B gene [15]. Many lasso peptide gene clusters also contain a gene encoding for an ABC transporter, which is presumably involved in the secretion of the lasso peptide. In other cases, a gene encoding for an isopeptidase is found in place of the ABC transporter. It has been proposed that clusters containing ABC transporters might encode lasso peptides with antimicrobial activity [2], whereas clusters containing isopeptidases might produce lasso peptides with other functions [16].

RES-701-3 and RES-701-4 (RES-701-3, -4) are two class II lasso peptides originally identified in the fermentation broth of *Streptomyces* sp. RE-896 while screening for endothelin antagonists [17,18,19]. Both compounds were shown to inhibit the endothelin 1 (ET-1) binding to the endothelin type B receptor (ET_B_), with IC_50_ values around 5–10 nM. Antagonists of ET-1 have been proposed as potential candidates for the treatment of diseases such as systemic hypertension, myocardial infarction, cardiac ischemia and diabetes mellitus, and numerous microbial natural products have been isolated during screening programs aiming to identify endothelin antagonists [20,21,22,23].

**Figure 1:**
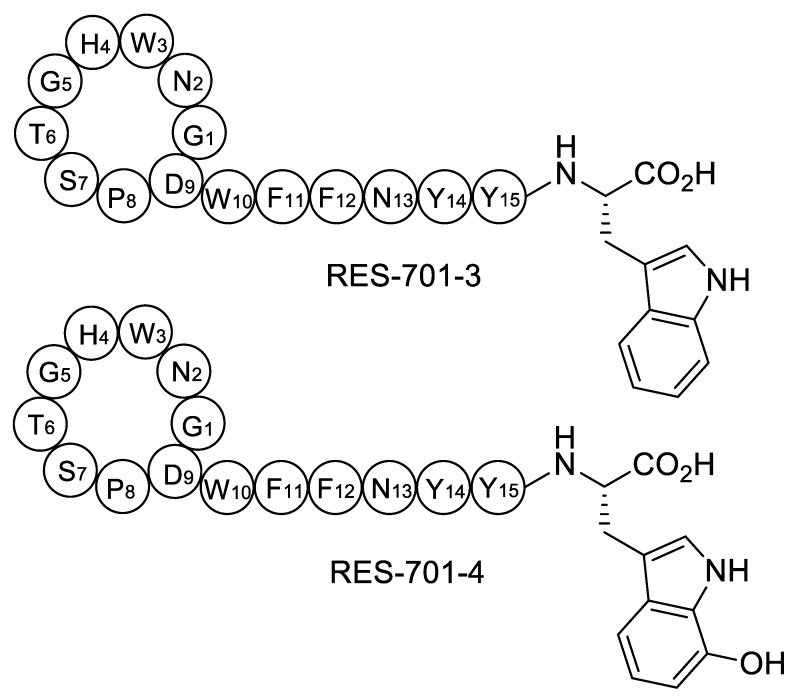
Structures of RES-701-3, -4 (pre-lasso conformation).

Chemical structures have been proposed for RES-701-3, -4, and they only differ in the identity of the C-terminal residue, which is tryptophan in RES-701-3 and 7-hydroxy-tryptophan in RES-701-4 (Figure 1) [17]. Their structurally related congeners RES-701-1 and RES-701-2 share this structural feature, with RES-701-1 containing C-terminal tryptophan and RES-701-2 containing 7-hydroxy-tryptophan [18,19]. It is reasonable to assume that the hydroxylation of position 7 of the C-terminal tryptophan in RES-701-1 and RES-701-3 leads to RES-701-2 and RES-701-4, respectively. Such hydroxylation is unlikely to happen during the isolation/purification of the compounds, and therefore is likely to occur enzymatically during the biosynthesis of the lasso peptides. In this study, we describe the identification, analysis, cloning and heterologous expression of the biosynthetic gene cluster directing RES-701-3 and -4 biosynthesis from the marine *Streptomyces caniferus* CA-271066. 

## 2. Results

### 2.1. Identification and in Silico Analysis of RES-701-3, -4 Biosynthetic Gene Cluster

**Figure 2:**
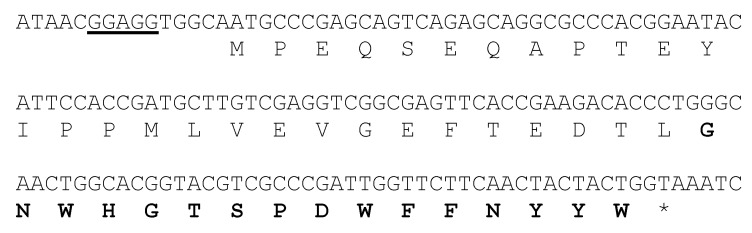
Nucleotide and deduced amino acid sequence of the *resA* region. A putative ribosome-binding sequence is underlined and the structural peptide for RES-701-3, -4 is shown in bold characters.

During our ongoing research with marine microorganisms, we identified *Streptomyces caniferus* CA-271066 as a producer of new bioactive metabolites [24]. The draft genome of this organism was analyzed with antiSMASH [10], which predicted 34 putative regions putatively encoding secondary metabolite gene clusters, including NRPS (non-ribosomal peptide synthetase), type I and II PKS (polyketide synthase), siderophores, terpenes and RiPPs. Careful examination of one of the RiPP gene clusters strongly suggested that it directed the biosynthesis of at least RES-701-3, based on the amino acid sequence predicted to be encoded by the precursor peptide gene *resA* (Figure 2).

**Figure 3:**
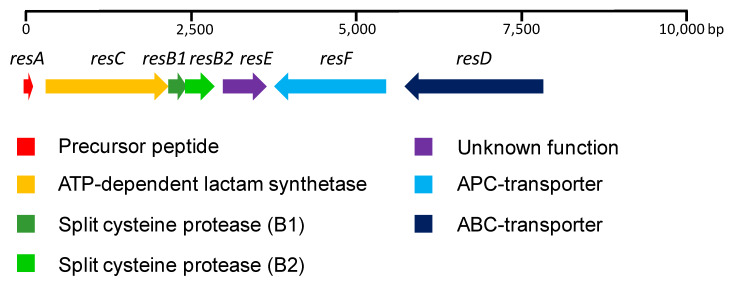
RES-701-3, -4 gene cluster from *Streptomyces caniferus* CA-271066.

A sequence analysis revealed the presence of seven ORFs located in a 7.7 Kb region (Figure 3). Interestingly, five of the ORFs (*resA*, *resC*, *resB1*, *resB2* and *resE*) are unidirectionally transcribed, whereas the remaining two (*resF* and *resD*) are transcribed from the complementary strand. Additionally, *resB1* and *resB2* are probably translationally coupled. *resA* encodes a 44 aa (amino acid) precursor peptide, with the C-terminal 16 aa region containing the core peptide and the remaining 28 aa from the N-terminal region forming the leader peptide required for processing. A BLASTp homology search using the NCBI non-redundant protein sequence database was employed to analyze the proteins encoded by the remaining ORFs. *resC* encodes a 612 aa protein, which was found to be similar to SOE12204.1 (630 aa, 84% identity, 89% similarity) from *Streptomyces* sp. 2323.1. The protein contains a cd01991 domain, which is typically found in the asparagine synthase and ATP-dependent lactam synthetases. *resB1* encodes an 84 aa protein similar to WP_106430390.1 (86 aa, 89% identity, 90% similarity) from *Streptomyces auratus*, and shows homology with the lasso peptide biosynthesis PqqD (pyrroloquinoline quinone biosynthesis) family chaperone. *resB2* encodes a 145 aa protein similar to SOE12206.1 (145 aa, 88% identity, 92% similarity) from *Streptomyces* sp. 2323.1, containing the domain pfam13471. *resE* encodes a 209 aa hypothetical protein similar to WP_006604205.1 (209 aa, 86% identity, 91% similarity) from *Streptomyces auratus*, and lacks any known conserved domain. *resF* encodes a 551 aa protein similar to WP_119203701.1 (551 aa, 88% identity, 94% similarity) from *Streptomyces* sp. 2233, it contains a COG0531 domain and is proposed to be a member of the APC (Amino Acid-Polyamine-Organocation) family of transporters. Its three-dimensional structure is predicted to contain 14 transmembrane helices (TMHMM server v 2.0) [25]. *resD* encodes a 691 aa protein similar to WP_106430393.1 (648 aa, 78% identity, 82% similarity) from *Streptomyces auratus* with high homology to ABC transporters. It contains a COG1132 domain and its three-dimensional structure is predicted to contain six transmembrane helices [25].

The growth of *Streptomyces caniferus* CA-271066 did not lead to the production of RES-701-3, -4 under any of the fermentation conditions employed. Thus, we conceived a strategy based on heterologous expression in order to establish a link between the lasso peptides and their putative biosynthetic gene cluster.

### 2.2. Cloning of resACB1B2EFD into the Vector pCAP01

A genomic region spanning 9.1 Kb and containing *resACB1B2EFD* was amplified by employing an overlapping-PCR approach. Over 700 nucleotides upstream of *resA* and *resD* were included in the amplified region in order to capture the putative promoter and the transcriptional and ribosome-binding sites. The 9.1 Kb SpeI/XhoI fragment was initially cloned into the pCR^TM^-Blunt vector and transformed into NEB 10-beta *E. coli*. Clones were checked by restriction analysis, and a digested and purified SpeI/XhoI fragment was then cloned into the pCAP01 vector, a *S. cerevisiae*/*E. coli*/actinobacteria shuttle vector designed for the site-specific integration of the cloned gene cluster into the chromosomes of heterologous actinobacterial hosts, thanks to the φC31 integration element present in the vector backbone, to generate pCAPRES [26]. Because pCAPRES contains a kanamycin-resistant marker, the direct transformation of the non-methylating CmR KmR *E. coli* strain ET12567/pUB307 is not possible. Thus, pCAPRES was used to transform the CmR *E. coli* strain ET12567, followed by triparental intergeneric conjugation employing *E. coli* ET12567/pCAPRES, ET12567/pUB307, and spores of the actinomycete host (*Streptomyces coelicolor* M1152, M1154, or *Streptomyces albus* J1074). Exconjugants were checked by PCR to confirm the integration of the putative biosynthetic gene cluster into the chromosomes of the heterologous hosts.

### 2.3. Heterologous Expression of RES-701-3, -4 Gene Cluster and LC-ESI-TOF MS Analysis

**Figure 4:**
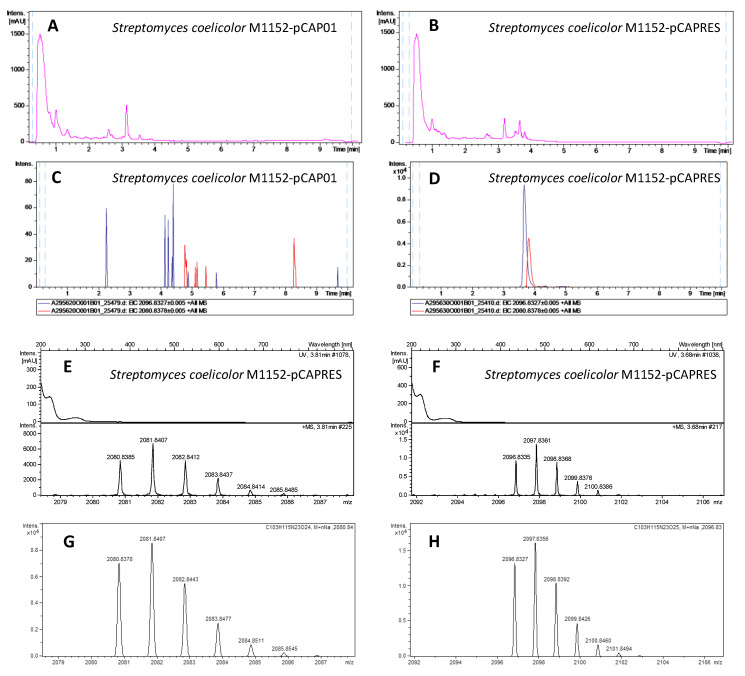
Heterologous expression of the RES-701-3, -4 gene cluster in *Streptomyces coelicolor* M1152. UV profiles at 210 nm of the negative control M1152-pCAP01 (**A**) and the positive exconjugant M1152-pCAPRES (**B**). Extracted Ion Chromatograms at 2080.8378 ± 0.005 (red trace, C_103_H_115_N_23_O_24_Na^+^, RES-701-3) and 2096.8327 ± 0.005 (blue trace, C_103_H_115_N_23_O_25_Na^+^, RES-701-4) in the extracts from the negative control M1152-pCAP01 (**C**, maximum intensity at around 80 counts) and the positive exconjugant M1152-pCAPRES (**D**, maximum intensity at around 10,000 counts). Zoomed-in image of the experimental MS isotopic patterns for the adducts C_103_H_115_N_23_O_24_Na^+^ (**E**) and C_103_H_115_N_23_O_25_Na^+^ (**F**) from the extract of the positive exconjugant M1152-pCAPRES. Theorical isotopic patterns for the adducts C_103_H_115_N_23_O_24_Na^+^ (**G**) and C_103_H_115_N_23_O_25_Na^+^ (**H**).

Positive exconjugants from *Streptomyces coelicolor* M1152, M1154 and *Streptomyces albus* J1074, together with negative controls (M1152, M1154 and J1074 conjugated with the empty vector pCAP01) were grown on five solid media (ISP2, ISP4, MYM, Minimal Medium and Supplemented Minimal Medium) for 6 days at 28 °C. The agar was then sliced and subjected to overnight n-butanol extraction. The solvent was removed, the residue resuspended in 20% DMSO/water and analyzed by LC-HRESI-TOF (liquid chromatography-high resolution electrospray ionization-time of flight). The extracts from exconjugants M1152-pCAPRES and M1154-pCAPRES showed two new peaks at 3.68 and 3.81 min with *m*/*z* values of 2096.8335 and 2080.8385 respectively, which were completely absent in the exconjugants M1152-pCAP01 and M1154-pCAP01 employed as negative controls (Figure 4). The component with mass 2096.8335 was assigned the molecular formula C_103_H_115_N_23_O_25_Na^+^ (calculated mass 2096.8326, 0.43 ppm error), corresponding to the sodium adduct of RES-701-4, while the component with mass 2080.8385 was assigned the molecular formula C_103_H_115_N_23_O_24_Na^+^ (calculated mass 2080.8377, 0.38 ppm error), corresponding to the sodium adduct of RES-701-3. The experimental isotopic patterns obtained for both components are in perfect agreement with the theorical ones calculated for such molecular formulae. The production of both components was maximised in supplemented minimal medium for M1154-pCAP01 and ISP-2 for M1152-pCAPRES, although the components could be detected in all the media employed in these two heterologous hosts. In general, a higher production of RES-701-4 compared to RES-701-3 was observed for all the growth conditions. 

On the other hand, no production of either RES-701-3 or RES-701-4 could be detected in any of the exconjugant J1074-pCAPRES clones employed under any of the growth conditions used.

## 3. Discussion

Post-translational modifications are unusual in lasso peptides, although a few examples have been reported recently, including the C-terminal phosphorylation in paeninodin [27], citrullination in citrulassin A [14], acetylation in albusnodin [28], C-terminal methylation of lassomycin and lassomycin-like lasso peptides [29,30], and the epimerization of the α-carbon from the C-terminal amino acid residue in MS-271 [31]. In this work, we prove that *resACB1B2EFD*, identified through a genome-mining approach, is sufficient for the biosynthesis of the lasso peptide RES-701-3 and its 7-hydroxy-tryptophan homologue RES-701-4 employing *Streptomyces coelicolor* M1152 and M1154 as heterologous hosts. No production of either lasso peptide could be detected in any of the pCAPRES exconjugants from *Streptomyces albus* under any of the growth conditions, which shows the importance of employing different heterologous hosts, even if they are from the same genus. The heterologous expression of a lasso peptide in *Streptomyces coelicolor* and a lack of production in *Streptomyces albus* has also been observed in the case of albusnodin [28].

Based in their homologies and the identified conserved domains, plausible biosynthetic roles for proteins ResA, ResB1, ResB2 and ResC can be proposed. *resA* encodes the precursor peptide. *resB1* and *resB2* encode for a “split” B protein; therefore, ResB1 is proposed to recognise and bind the leader peptide from ResA to deliver the structural peptide to ResB2 for processing [15]. *resC* encodes for a lactam synthetase, which is proposed to catalyze the ATP-dependent formation of the amide bond between the N-terminal α-amino group of Gly-1 and the β-carboxyl group of Asp-8 [32].

Many lasso peptide BGCs contain an ABC transporter, presumably involved in the secretion of the mature product, which is usually found downstream of the lactam synthetase-encoding gene. In the RES-701-3, -4 gene cluster *resD* encodes for an ABC-type transporter and is located in the complementary strand to *resACB1B2E*. *resF* is adjacent to *resD* and encodes for a 551 aa protein with 14 transmembrane helices that belongs to the Amino Acid-Polyamine-Organocation (APC) family of transporters. To the best of our knowledge, this is the first time that a member of this family of transporters has been found in a lasso peptide biosynthetic gene cluster, and is not clear what role, if any, it could play in RES-701-3, -4 biosynthesis. Presumably, the concerted action of ResD with additional ABC transporter components encoded elsewhere in the genome could be responsible for RES-701-3, -4 secretion.

**Figure 5:**
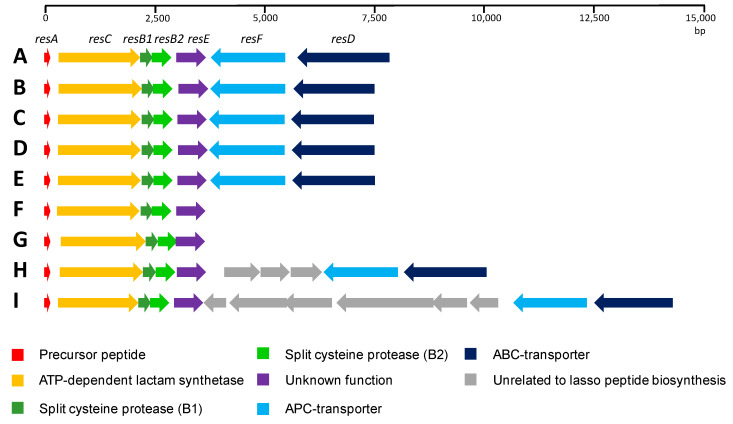
Comparison of the RES-701-3, -4 gene clusters found using a BLASTP homology search of ResE. *Streptomyces caniferus* CA-271066 (A); *Streptomyces sp*. 2314 (B); *Streptomyces sioyaensis* (C); *Streptomyces sp*. 2333.5 (D); *Streptomyces sp*. 2112.2 (E); *Streptomyces auratus* (F); *Streptomyces angustmyceticus* (G); *Streptomyces* TM32 (H); and *Streptomyces sp*. 2323.1 (I).

On the other hand, *resE* is located downstream of *resB2*, and it encodes a medium-sized protein (209 aa), lacking any known conserved domain. Close homologues of ResE are found in the genomes of eight other *Streptomyces* strains. An analysis of the genomic context for these ResE analogues shows that all of them are encoded within homologous RES-701-3, -4 gene clusters (Figure 5), strongly suggesting that ResE is required for the biosynthesis of these lasso peptides.

A close inspection of the ResA protein shows that the C-terminal 16 aa region containing the structural peptide is identical in all the cases, and only minor differences are found in the N-terminal region, corresponding to the leader peptide. In four of the cases, *Streptomyces sp.* 2314.4, *Streptomyces sioyaensis*, *Streptomyces* sp. 2333.5 and *Streptomyces* sp. 2112.2, the gene cluster organization is identical to that described here for *Streptomyces caniferus* CA-271066. *Streptomyces auratus* and *Streptomyces angustmyceticus* NRRL B-2347 contain the operon *resACB1B2E* and lack the genes *resD* and *resF*. Finally, in the case of *Streptomyces* sp. TM32 and *Streptomyces* sp. 2323.1 a number of ORFs encoding for proteins unrelated to lasso peptide biosynthesis are found embedded between *resF* and *resD*. These data suggest that ResD and ResF might not be strictly required for RES-701-3, -4 biosynthesis. On the other hand, it is worth mentioning that in the case of *Streptomyces angustmyceticus* NRRL B-2347, *resB2* and *resE* might be translationally coupled, which could suggest a coordinated action and/or protein–protein interaction between ResB2 and ResE [33]. More distantly related homologues of ResE can be found in the genomes of numerous other *Streptomyces* species, but there is a noticeable drop in the level of homology and an analysis of their genomic context shows that they are not encoded within lasso peptide gene clusters.

The fact that i) *resACB1B2EFD* is sufficient to produce both lasso peptides, ii) putative roles for ResA, ResC, ResB1 and ResB2 can be proposed based in their homologies, iii) ResD and ResF are transmembrane transporters and, as such, are unlikely to be involved in the conversion of RES-701-3 into RES-701-4 and iv) all of the closest homologues of ResE are encoded in homologous RES-701-3 or -4 gene clusters found in eight other *Streptomyces* species, lead us to hypothesise that ResE is likely to play a key role in the hydroxylation of position 7 of the C-terminal tryptophan of RES-701-3 or its pre-lasso intermediate in order to afford RES-701-4.

In summary, we report the identification, cloning and heterologous expression of the gene cluster encoding the biosynthesis of RES-701-3, -4. Our data unequivocally shows that *resACB1B2EFD* is sufficient for the production of both lasso peptides. Additionally, genome mining allowed us to identify eight other *Streptomyces* strains potentially containing the RES-701-3, -4 biosynthetic gene cluster, in which *resE* is universally conserved. We hypothesise that ResE is likely to play a key role in the hydroxylation required to generate RES-701-4, but further genetic and/or biochemical characterization work will be required to test this hypothesis and decipher the exact roles of ResE and ResF in the biosynthesis of these two lasso peptides.

## 4. Materials and Methods 

### 4.1. Bacterial Strains and Plasmids

The strain *Streptomyces caniferus* CA-271066 was isolated from an ascidian collected at the seaside at 2 meters depth in São Tomé (São Tomé and Principe). A similarity-based search with the 16S rDNA sequence (1393 nt) against the EzBioCloud database indicated that the strain is closely related to *Streptomyces caniferus* DSM 41453(T) (100% identity) [34]. NEB 10-beta competent *E. coli* (New England BioLabs, Ipswich, MA, USA), *E. coli* ET12567 (LGC Standards, Manchester, NH, USA) and *E. coli* ET12567/pUB307 (kindly provided by J.A. Salas) were employed throughout the cloning and conjugation processes, together with the vectors pCR^TM^-Blunt (Thermo Fisher Scientific, Waltham, MA, USA) and pCAP01 (provided by Bradley Moore (Addgene plasmid #59981; http://n2t.net/addgene:59981; RRID: Addgene_59981)) [26]. *Streptomyces coelicolor* M1152 and M1154 [35] were generously provided by M. Bibb. *Streptomyces albus* J1074 [36] was generously provided by J. A. Salas.

### 4.2. Growth and Culture Conditions

*Streptomyces caniferus* CA-271066 was typically cultured on ATCC-2 medium (soluble starch 20 g/L, glucose 10 g/L, NZ Amine Type E 5 g/L, meat extract 3 g/L, peptone 5 g/L, yeast extract 5 g/L, calcium carbonate 1 g/L, pH 7) and grown on an orbital shaker at 28 °C, 220 rpm and 70% relative humidity. *E. coli* strains were routinely cultured in LB Miller broth (Sigma-Aldrich, St. Louis, MO, USA) (37 °C, 250 rpm) and Difco LB Lennox agar (37 °C, static). Intergeneric conjugations were carried out on MA (MOPS 21 g/L, glucose 5 g/L, yeast extract 0.5 g/L, beef extract 0.5 g/L, casamino acids 1 g/L, agar 25 g/L, pH adjusted to 7). Exconjugants were grown on ISP2, ISP4, minimal medium, supplemented minimal medium [37] and MYM (maltose 4 g/L, yeast extract 4 g/L, malt extract 10 g/L, tap water 0.5 L, milli-Q water 0.5 L; once autoclaved add 2 mL of R2 trace elements stock). Antibiotics were supplemented when required for the selection of transformants at the following final concentrations: kanamycin (50 μg/mL), nalidixic acid (25 μg/mL), choramphenicol (25 μg/mL).

### 4.3. General Molecular Biology Techniques

Restriction endonucleases, Q5 High-Fidelity polymerase and the T4 DNA Ligase (Blunt/TA Ligase Master Mix) were purchased from New England Biolabs (Ipswich, MA, USA). Calf Intestinal Alkaline Phosphatase (CIAP) was purchased from Invitrogen (Waltham, MA, USA). All the primers employed were purchased at Sigma-Aldrich (St. Louis, MO, USA). A QIAprep Spin Miniprep Kit (Qiagen, Hilden, Germany) was employed for the purification of plasmid DNA from cells and the Illustra^TM^ GFX^TM^ PCR DNA and Gel Band Purification Kit (GE Healthcare, Boston, MA, USA) was used for the purification of DNA amplicons from agarose gels and enzymatic reactions.

### 4.4. PCR Amplifications of RES-701-3, -4 Biosynthetic Gene Cluster

All attempts to amplify a 9.1 Kb DNA fragment containing the biosynthetic gene cluster failed. A strategy based on the amplification of two overlapping regions was thus employed. Region A was amplified using primers resAF 5’-TAAGCAACTAGTGGTGGAAGCCCCCTTTGG-3’ (forward, SpeI restriction site underlined) and resAR 5’-GACTCAGGTCCCGCCCC-3’ (reverse) on 50 μL PCR mixtures containing 10 μL Q5 buffer (5×), 0.2 mM dNTPs, 0.5 μM forward and reverse primers, 1 μL of template DNA, 10 μL of enhancer and 0.5 μL of Q5 polymerase. The PCR conditions were 98 °C for 30 s, followed by 35 cycles at 98 °C for 10 s, 70 °C for 30 s, 72 °C for 3.5 min, with a final elongation step at 72 °C during 5 min. Region B was amplified using primers resBF 5’-CAGCGTTCGACAGCCTGG-3’ (forward) and resBR 5’-TAAGCACTCGAGCAACGCTGTGTGAGGCCA-3’ (reverse, XhoI restriction site underlined) on 50 μL PCR mixtures containing 10 μL Q5 buffer (5×), 0.2 mM dNTPs, 0.5 μM forward and reverse primers, 1 μL of template DNA, 10 μL of enhancer and 0.5 μL of Q5 polymerase. The PCR conditions were 98 °C for 30 s, followed by 35 cycles at 98 °C for 10 s, 65 °C for 30 s, 72 °C for 3.5 min, with a final elongation step at 72 °C during 5 min. Region A and region B amplicons were purified and employed in a two-step overlapping PCR with 50 μL mixtures containing 10 μL Q5 buffer, 0.4 mM dNTPs, 10 μL of enhancer, equimolecular amounts of the region A and B amplicons (ca. 500 ng), and 0.5 μL of Q5 polymerase. The PCR conditions were 98 °C for 30 s, followed by 15 cycles at 98 °C for 10 s, 65 °C for 30 s, 72 °C for 3.5 min, with a final elongation step at 72 °C during 5 min. Once finished, primers resAF and resBR were added at 0.5 μM final concentration and the second PCR step was carried out: 98 °C for 30 s, followed by 30 cycles at 98 °C for 10 s, 65 °C for 30 s, 72 °C for 8 min, with a final elongation step at 72 °C during 15 min. The overlapped 9.1 Kb amplicon obtained was purified prior to its cloning.

### 4.5. Cloning of RES-701-3, -4 Biosynthetic Gene Cluster into the pCPA01 Vector

The 9.1 Kb amplicon containing RES-701-3, -4 gene cluster was initially cloned into the pCR^TM^-Blunt vector using the Zero Blunt^TM^ PCR Cloning kit (Thermo Fisher Scientific, Waltham, MA, USA). Briefly, ca. 975 ng of the 9.1 Kb amplicon was mixed with 0.5 μL of the pCR^TM^-Blunt vector, 1.5 μL of the ligase buffer, 1.5 μL of the T4 DNA ligase and the mixture was incubated at room temperature during 90 min. A total of 10 μL of the mixture were then used to transform NEB 10-beta competent *E. coli* (New England BioLabs, Ipswich, MA, USA). The generated recombinant plasmids (pBLUNT-RES) were confirmed by a restriction analysis. pBLUNT-RES was digested with SpeI and XhoI and the resulting 9.1 Kb fragment was purified and ligated by T4 DNA ligase with the largest dephosphorylated SpeI/XhoI fragment of pCAP01, followed by the transformation of NEB 10-beta competent *E. coli*. The recombinant plasmids were analyzed by restriction digestion to obtain pCAPRES.

### 4.6. Intergeneric Conjugation

Plasmid pCAPRES was conjugated into *Streptomyces* hosts as previously described [26]. Briefly, purified pCAPRES was used to electroporate non-methylating *E. coli* ET12567. Cells from *E. coli* ET12567/pUB307 and *E. coli* ET12567/pCAPRES collected at an optical density of 0.4–0.6 were washed with LB twice to remove the antibiotics, resuspended and mixed with an adequate amount of freshly activated spores of *S. coelicolor* M1152, M1154 and *S. albus* J1074. The mixtures were plated on MA for triparental mating and overlaid after ca. 16 h with nalidixic acid (25 μg/mL) and kanamycin (50 μg/mL). After a few days of incubation, some of the exconjugants were streaked on MA plates containing nalidixic acid (25 μg/mL) and kanamycin (50 μg/mL) and five colonies from each *Streptomyces* heterologous host were picked and streaked on ISP-2 plates. The insertion of pCAPRES into the *Streptomyces* hosts chromosomes was checked by PCR employing the genomic DNA of the exconjugants and the primers resBF and resBR.

### 4.7. Heterologous Expression of RES-701-3, -4 Gene Cluster and LC-ESI-TOF Analysis

Seed cultures on ATCC-2 of the recombinant strains *S. coelicolor* M1152, M1154 and *S. albus* J1074 harbouring RES-701-3, -4 BGC, together with the corresponding negative controls, were used to inoculate Petri plates with ISP2, ISP4, MYM, minimal medium and supplemented minimal medium. The plates were incubated at 28 °C for 6 days and then the agar was sliced and subjected to extraction with n-BuOH. The organic solvent was evaporated to dryness and the extract was resuspended to a final ratio of 20% DMSO/water. The microbial extracts were filtered and analyzed employing a Bruker maXis QTOF mass spectrometer coupled to a HPLC system, as previously described [38].
